# Alveolar ridge preservation with a collagen cone: Histological, histochemical, and immunohistochemical results of a randomized controlled clinical trial

**DOI:** 10.1002/cre2.279

**Published:** 2020-01-22

**Authors:** Sigmar Schnutenhaus, Cornelia Edelmann, Jens Dreyhaupt, Heike Rudolph, Ralph G. Luthardt, Werner Goetz

**Affiliations:** ^1^ Center of Dentistry Dr. Schnutenhaus MVZ GmbH Hilzingen Germany; ^2^ Department of Prosthetic Dentistry, Center of Dentistry Ulm University Ulm Germany; ^3^ Institute of Epidemiology and Medical Biometry Ulm University Ulm Germany; ^4^ Department of Orthodontics, Oral Biology Laboratory University of Bonn Bonn Germany

**Keywords:** alveolar ridge preservation, bone regeneration, collagen, extraction sockets, histochemistry, immunohistochemistry

## Abstract

**Objectives:**

The objective of the present study was to examine the influence of a combination material of a collagen cone and a collagen membrane on the healing process of extraction sockets with regard to histological, histochemical, and immunohistochemical parameters.

**Materials and methods:**

In a prospective randomized clinical study, 10 patients (test group) received a collagen combination material after tooth removal. The extraction sockets of 10 other patients (control group) were left to heal without further intervention. Eleven ±1 weeks after tooth extraction, histological biopsies were performed in both groups at the time of implant placement. Subsequently, the biopsies were evaluated semiquantitatively in terms of histological, histochemical, and immunohistochemical parameters for the identification of factors of bone metabolism and vascularization.

**Results:**

No significant difference between test and control group were found for any parameter. According to the descriptive data, the use of a collagen combination material seems to result in slightly higher values of the osteogenic Runt‐related transcription factor 2 (Runx2) and vascularization.

**Conclusion:**

The histological, histochemical, and immunohistochemical analysis of ARP with a collagen cone combined with a collagen membrane showed no significant differences in terms of bone metabolism and vascularization.

## 1 INTRODUCTION

Resorptive changes of the alveolar process are observed after tooth extraction (Tan, Wong, Wong, & Lang, 2012). The loss of bone volume has a great influence on the therapeutic effort and the result of implant therapy following tooth extraction. Position, angulation, and therefore the prognosis of the implant are dependent on the available soft and hard tissue (Karaca, Er, Gülşahı, & Köseoğlu, 2015). The natural regeneration after tooth loss starts with the formation of a blood coagulum, which contains fibrin as the leading structure for new bone development (Schmidlin, Jung, & Schug, 2004). Osteogenesis starts with islands of bone within the connective tissues 4–8 weeks after tooth removal (Trombelli et al., 2008). More developed trabecular structures and less osteoblasts can be seen at 10–12 weeks (Evian, Rosenberg, Coslet, & Corn, 1982). After 120 days, new bone formation is concluded, followed by the complete formation of periosteum after approximately 180 days (Evian et al., 1982; Iyer, Haribabu, & Xing, 2014). New bone formation does not lead to restitutio ad integrum. Due to bone loss after tooth extraction within the first 6 months, the alveolar process is being reduced by an average of 3.8 mm horizontally and 1.2 mm vertically (Masaki, Nakamoto, Mukaibo, Kondo, & Hosokawa, 2015; Pagni et al., 2012).

The loss of bone volume can affect the surgical effort in terms of necessary grafting measures and consequently higher treatment costs. Another aspect of horizontal and vertical bone loss is the chance of compromising functional and esthetic results (Chappuis, Araujo, & Buser, 2017; Kesmas, Swasdison, Yodsanga, Sessirisombat, & Jansisyanont, 2010).

To prevent bone loss, different actions after tooth extraction are taken to influence bone and soft tissue healing. Clinical concepts like the insertion of different bone substitutes, the closure of the empty socket with a membrane (Barone et al., 2008; Cardaropoli, Tamagnone, Roffredo, Gaveglio, & Cardaropoli, 2012; Ten Heggeler, Slot, & Van der Weijden, 2011), and the plastic covering with an advancement flap or a gingival transplant (Fickl et al., 2011) are being proposed. These measures of alveolar ridge preservation (ARP) show significant positive effects on bone loss (Avila‐Ortiz, Elangovan, Kramer, Blanchette, & Dawson, 2014; Bassir et al., 2018; Horowitz, Holtzclaw, & Rosen, 2012). Implants being placed in regenerated parts of the alveolar ridge show similar survival rates to implants being placed in natural bone, but it was not possible to demonstrate that one augmentation technique is superior compared to another technique based on implant survival rates (Chen et al., 2009; Corbella, Taschieri, Francetti, Weinstein, & del Fabbro, 2017). There was also no evidence for the superiority of one type of ARP intervention with regard to the formation of new bone (Corbella et al., 2017; MacBeth, Trullenque‐Eriksson, Donos, & Mardas, 2017) or bone dimensional preservation and keratinized tissue dimensions (MacBeth et al., 2017).

The reduction of bone resorption with the use of a completely absorbable material is an innovative and promising concept. Extraction sockets that received a collagen cone combined with a collagen membrane after tooth removal in an animal study showed a significant reduction of bone resorption compared to extraction sockets without intervention (Kunert‐Keil et al., 2015).

Parasorb Sombrero® (Resorba, Nürnberg, Germany) is a new, completely resorbable material containing the combination of a collagen cone with equine collagen fibrils of Type I and a collagen membrane. To ensure simple and quick utilization, one product combines these two materials.

Up to now, there are no sufficient clinical trials on humans analyzing the material combination of a collagen cone with a collagen membrane (Annen, Schneider, & Schmidlin, 2014; Kunert‐Keil et al., 2015). In a recent clinical and histomorphometrical study, we have shown that there are no significant differences in terms of new bone formation and bone quality when performing ARP with Parasorb Sombrero® after tooth removal compared to a control group without ARP (Schnutenhaus, Götz, Dreyhaupt, Rudolph, & Luthardt, 2018). Descriptively, however, different findings like increased bone remodeling, increased osteoblast activity, and increased vascularization were seen (Schnutenhaus, Götz, et al., 2018).

However, although histology and histomorphometry can indicate structural changes within the augmented socket, for example, osteogenesis or inflammation, the biological processes behind these changes remain elusive. Immunohistochemistry investigations allow to localize factors involved in these processes and to draw conclusions about the biological functions on cellular and even molecular levels. Only a few immunohistochemical studies have been undertaken in biopsies from patients after augmentation of sockets. Most of them have focused on the remodeling of deproteinized bovine bone by using anabolic and catabolic bone markers (e.g., Milani, Dal Pozzo, Rasperini, Sforza, & Dellavia, 2016).

The aim of this study was to examine the influence of a combination material of a collagen cone and a collagen membrane on the healing process of extraction sockets with regard to histological, histochemical, and immunohistochemical parameters.

## 2 MATERIALS AND METHODS

The study was conducted as a prospective controlled randomized clinical study according to the Declaration of Helsinki. The procedure and all the materials used were submitted to the relevant Ethics Committee of the University of Ulm and approved (No. 337/12, approved February 13, 2013).

The study participants were informed about the study before their participation, both orally and in writing, and gave their written informed consent. The study design corresponds to the previously published article of Schnutenhaus, Götz, et al. (Schnutenhaus, Götz, et al., 2018).

### 2.1 Study population

Twenty patients with at least one tooth in the upper jaw that had to be removed and to be replaced by a fixed implant‐supported restoration participated in the study. Two groups (test and control groups) of 10 patients each from a study with a total of 60 participants were included in consecutive order (Schnutenhaus et al., 2017).

Ten patients received ARP after tooth extraction (test group). In the other 10 patients, wound healing was allowed to proceed without further intervention. Inclusion and exclusion criteria are listed in detail in the previously published article of Schnutenhaus, Götz, et al. (Schnutenhaus, Götz, et al., 2018).

No differentiation was made in the indication of whether the tooth had to be extracted for periodontal reasons, carious destruction, or trauma, for example.

### 2.2 Treatment protocol

The study took place in the private practice of the first author (S. I. S.), who exclusively performed all interventions and follow‐ups. All participating patients were recruited in the same private practice in Hilzingen, Germany.

**Figure 1:**
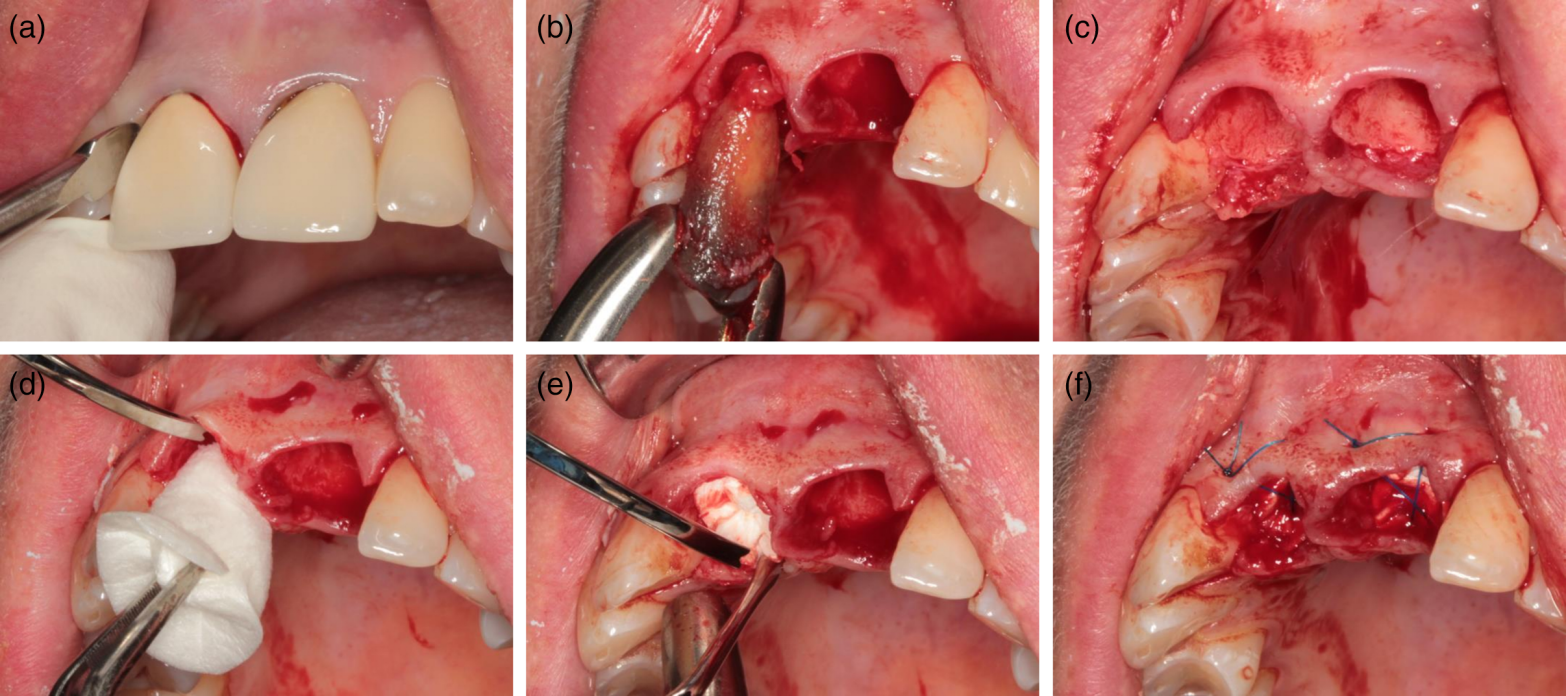
(a, b) Atraumatic tooth removal with periotoms and forceps. (c) Extraction sockets after careful curettage. (d, e) Introducing of the collagen cone with membrane. (f) Sutures to stabilize the membrane

Interventions on the day of tooth extraction included local anaesthesia with articaine (Ultracain DS 1:200,000; Sanofi Aventis, Frankfurt, Germany), gentle extraction of the teeth after complete mobilization, curettage of the extraction socket, and no further measures for the patients of the control group. The patients of the test group received a combination material consisting of a collagen cone and a collagen membrane according to the manufacturer's instructions (Figure 1a–f). All patients were instructed about the behavior for the following 24 hr after tooth removal. One week later, visual inspection of all wounds was performed.

A nonsteroidal anti‐inflammatory drug (600‐mg ibuprofen) was prescribed.

Patients who needed a provisional removable restoration for either esthetic or functional reasons or at their personal request were given an interim prosthesis. Implant positions were determined by using an implant planning software (SMOP; Swissmeda, Zürich, Switzerland) and consequently transferred by means of a surgical template on the day of implantation which was 11 ± 1 weeks after tooth removal.

A trephine drill was used for sample collection at implant site. The treatment protocol can be read in detail in the previously published article of Schnutenhaus, Götz, et al. (Schnutenhaus, Götz, et al., 2018).

### 2.3 Histology and histochemistry

The method of the histological analysis has been previously published in two studies by the last author (Friedmann, Gissel, Konermann, & Götz, 2015; Solakoglu, Götz, Heydecke, & Schwarzenbach, 2019). Each sample was fixed by immersion in 4% buffered formaldehyde (Sörensen buffer) at room temperature (RT) for at least 1 day and subsequently decalcified for about 2 to 3 weeks in 4.1% disodium ethylene‐diamino‐tetraacetic acid‐solution, which was changed every 24 hr. After hydration, tissues were dehydrated in an ascending series of ethanol and embedded in paraffin. Serial sagittal sections of 2–3 μm were cut, and representative slides were stained with haematoxylin‐eosin, Masson‐Goldner trichrome, and periodic acid–Schiff staining for histochemical detection of glycosaminoglycans and glycoproteins. In order to identify osteoclasts, selected tissue sections were stained to demonstrate tartrate‐resistant acid phosphatase.

### 2.4 Immunohistochemistry

Representative slides from the median parts of the sample series were deparaffinized, rehydrated, and rinsed for 10 min in tris‐buffered saline. Endogenous peroxidase was blocked in a methanol/H_2_O_2_ (Merck, Darmstadt, Germany) solution for 45 min in the dark. Sections were pretreated with phosphate‐buffered saline containing 1% bovine serum albumin for 20 min at RT, digested with 0.4% pepsin for 10 min at 37°C, and afterwards incubated with the primary antibodies in a humid chamber. Antibody details and incubation protocols are listed in Table 1. Detection of antibody binding was performed with the peroxidase‐conjugated EnVision® antimouse system or the EnVision® antirabbit/antigoat HRP‐conjugated secondary antibodies (horseradish peroxidase, Dako, Glostrup, Denmark) diluted 1:50 and incubated for 30 min at RT. Peroxidase activity was visualized using diaminobenzidine (DAB) yielding a brown staining product, and slides were counterstained with Mayer's haematoxylin.

**Table 1:** Antibody details and incubation protocols

Antibody	Isotype	Manufacturer	Incubation protocol
Alkaline phosphatase	Rabbit polyclonal	Quartett (Berlin, Germany)	Ready to use, on, 4°C
Collagen Type I	Mouse monoclonal	Abcam (Cambridge, UK)	1:200, 1 hr, rt
ED1 (CD 68)	Mouse monoclonal	Dako (Glostrup, Denmark)	1:100, 1 hr, rt
Osteocalcin	Mouse monoclonal	Takara (Otsu, Shiga, Japan)	1:100, 1 hr, rt
Osteopontin	Rabbit polyclonal	Abcam (Cambridge, UK)	1:200, 1 hr, rt
Runt‐related transcription factor 2 (Runx2)	Goat polyclonal	Santa Cruz (Santa Cruz, Ca, USA)	1:30, on, 4°C
von Willebrand factor	Rabbit polyclonal	Linaris (Wertheim, Germany)	1:200, 1 hr, rt

Abbreviation: rt, room temperature.

Specificity controls were run by (a) omitting primary antibodies and applying tris‐buffered saline or normal horse serum instead and (b) omitting primary antibodies or bridge and secondary antibodies. Mandibular bone or fetal human bone tissues carrying known antigens were used as positive controls.

### 2.5 Histological evaluation

The qualitative and semiquantitative evaluation of the histological sections was done on the basis of established scoring methods in bone histology and pathology (Fedchenko & Reifenrath, 2014) and methods applied to parameters investigated in similar studies on the healing of bone substitutes (Koerdt, Ristow, Wannhoff, Kübler, & Reuther, 2014; Konermann et al., 2016). The blinded evaluation was carried out by two experienced investigators in three different sections of the serial sections. Representative regions of interest were localized in the center of the section and in two apically, coronally, or laterally localized regions bordering the autochthonous bone tissue.

The semiquantitative evaluation of infiltrates was performed according to the following scheme:

0 = *no infiltrations*,

1 = *loose or disseminated occurring inflammatory cells, focally appearing*,

2 = *dense round cell infiltrates of moderate volumes*,

3 = *extended, dense round cell infiltrations with high endothelial venules, edema, focally multinucleated giant cells*, and

4 = *strong inflammatory reaction including giant cells and necrosis*.

The semiquantitative evaluation of histochemical (tartrate‐resistant acid phosphatase) and immunohistochemical (ED1, Runt‐related transcription factor 2 [runx2], and alkaline phosphatase) findings restricted to cellular reactions was done according to

0 = *negative*,

1 = *weak*,

2 = *moderate*,

3 = *strong*, and

4 = *very strong*.

For the evaluation of alkaline phosphatase, immunoreactivity within vessel walls was not considered.

The semiquantitative evaluation of von Willebrand factor (VWF) was carried out according to the density of immunoreactive vessel lumina or tangential wall cuts:

0 = *negative*,

1 = *weak*,

2 = *moderate*,

3 = *strong/dense*, and

4 = *very strong/dense*.

The semiquantitative evaluation of bone matrix protein appearing in a cellular but also extracellular (bone matrix, connective tissue matrix, etc.) manner (Type I collagen, osteocalcin [OC], and osteopontin [OP]) was performed according to the following scheme:

0 = *negative*,

1 = *immunoreactivity only in cells, for example, osteoblasts, and fibroblasts*,

2 = *immunoreactivity in cells as well as extracellularly in early osteogenesis (e.g., osteoid) or weakly in connective tissue areas*,

3 = *immunoreactivity in cells as well as moderately in bone matrix and connective tissue*, and

4 = s*trong immunoreactivity in both locations*.

**Figure 2:**
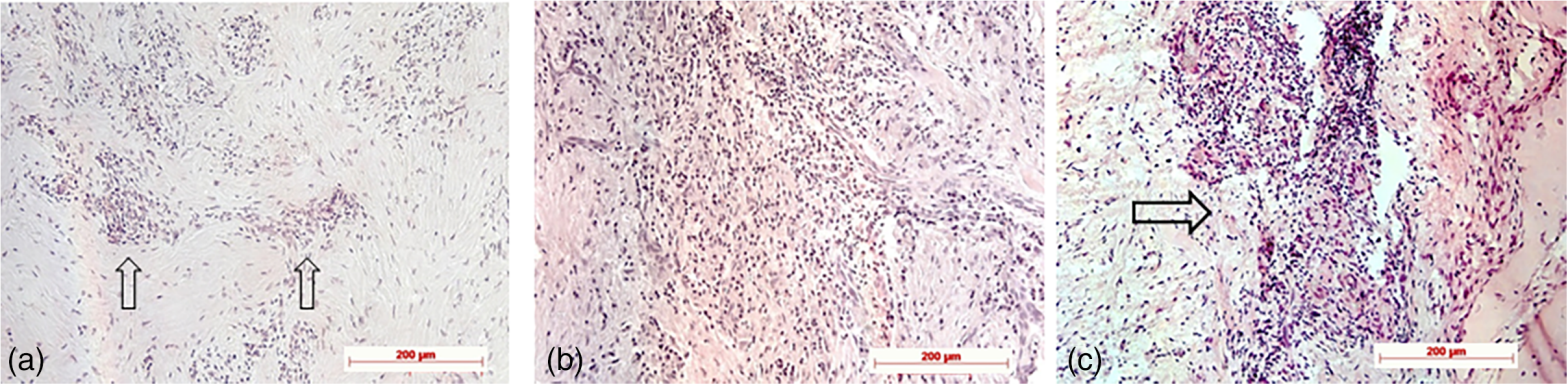
Infiltrates: (a) Stage 1: focal loose or disseminated occurring inflammatory cells (arrows), control specimen, H.E.; (b) Stage 2: dense, round cell infiltrate (center), test specimen, H.E.; and (c) Stage 3: extended, more dense round cell infiltrate with multinucleated giant cells (arrow), H.E., control specimen; original magnification all ×20

**Figure 3:**
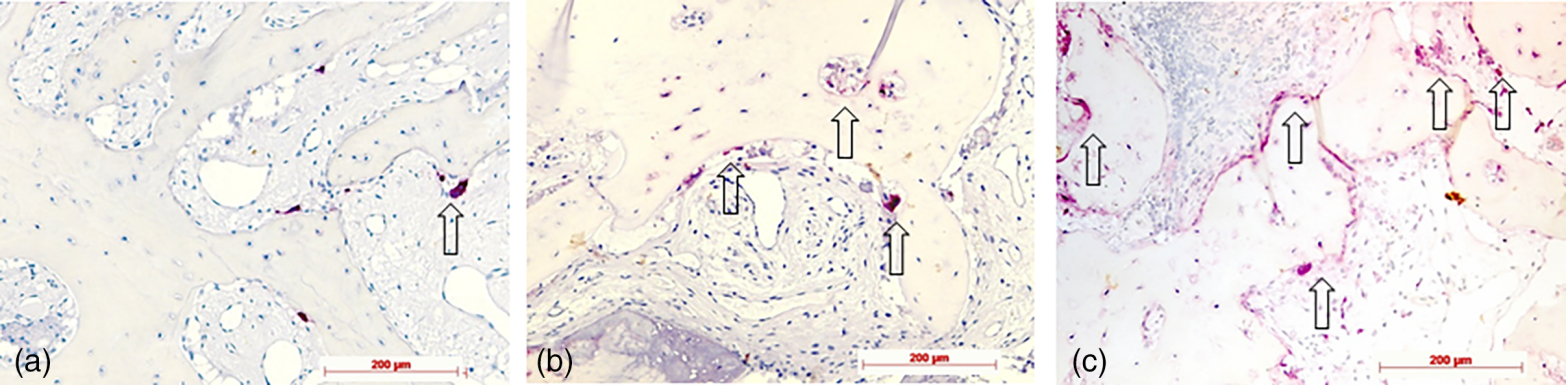
Tartrate‐resistant acid phosphatase (TRAP) staining for osteoclast identification: (a) Stage 1: weak (few TRAP+ cells), test specimen; (b) Stage 2: moderate occurring TRAP+ cells; control specimen; and (c) strong (high number of TRAP+ cells), test specimen; original magnification all ×20

**Figure 4:**
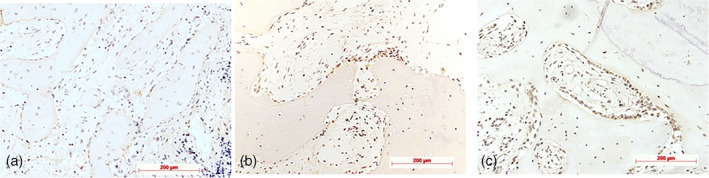
Runt‐related transcription factor 2 (Runx2) immunohistochemistry for detection of preosteoblasts: (a) Stage 1: weak (few Runx2+ cells on bone surface [arrow]), diaminobenzidine (DAB), control specimen; (b) Stage 2: moderate occurring Runx2+ cells (arrows: Runx2+ cells clustering on bone surface); DAB, control specimen; (c) strong (high number of Runx2+ cells, seams of Runx2+ cells on bone surfaces [arrows], and in intertrabecular tissue), DAB, test specimen; original magnification all ×20

**Figure 5:**
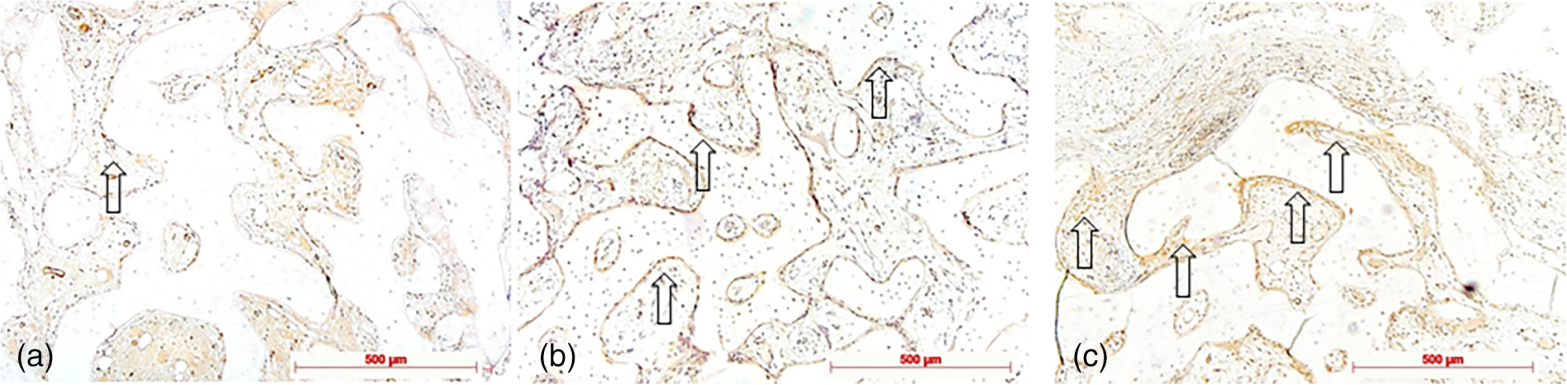
Alkaline phosphates (AP) immunohistochemistry for detection of osteoblasts and young osteocytes: (a) Stage 1: weak (few AP+ cells on bone surface [arrow]), diaminobenzidine (DAB), control specimen; (b) Stage 2: moderate occurring AP+ cells (arrows: AP+ cell seams on bone surface); DAB, control specimen; (c) strong (high number of AP+ cells, multilayered seams of AP2+ cells on bone surfaces [arrows]), DAB, control specimen; original magnification all ×10

**Figure 6:**
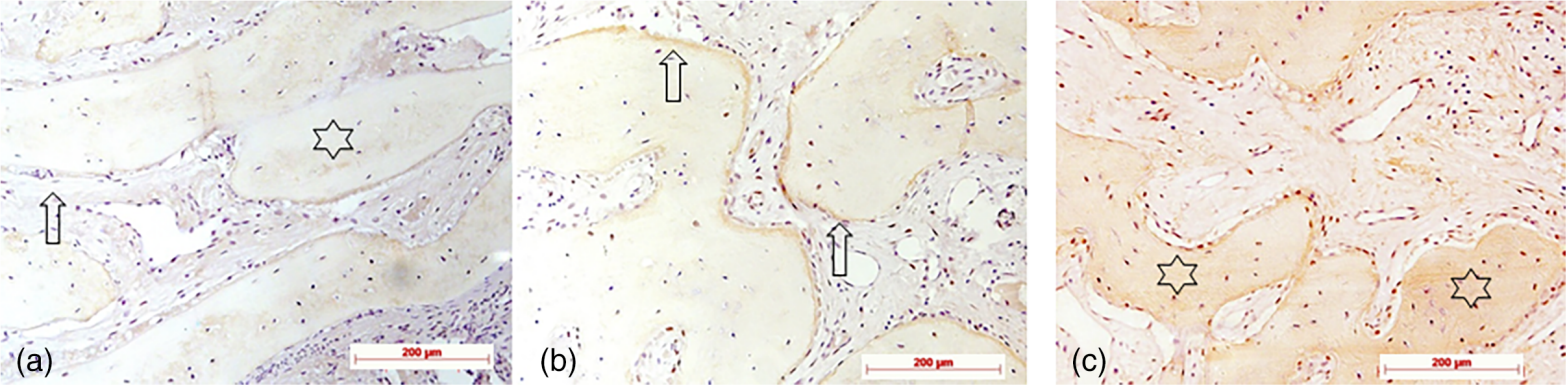
Von Willebrand factor (VWF) immunohistochemistry for detection of vessels: (a) Stage 1: weak vessel density indicated by only a few immunostained brownish profiles (lamina propria), diaminobenzidine (DAB), control specimen; (b) Stage 2: moderate vessel density indicated by immunostained brownish profiles (newly formed bone), DAB, test specimen; (c) Stage 3: higher vessel density indicated by immunostained brownish profiles (newly formed bone), DAB, test specimen; original magnification all ×10

**Figure 7:**
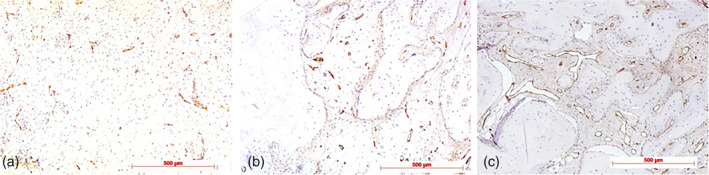
Collagen I immunohistochemistry in newly formed bone: (a) Stage 1: immunoreactivity in few osteoblasts or lining cells on bone surfaces (arrow), no or minimal staining of bone matrix (asterisk), diaminobenzidine (DAB), test specimen; (b) Stage 2: immunostaining in cells as well as extracellularly in osteoid (arrows); DAB, test specimen; (c) Stage 3: immunoreactivity in cells (arrow) as well as in bone matrix (asterisks), DAB, test specimen; original magnification all ×20

Histological, histochemical, and immunohistochemical examples for staging according to semiquantitative evaluation are demonstrated in Figures 2, 3, 4, 5, 6, 7.

### 2.6 Estimated sample size

Due to the lack of clinical data, no a priori sample size estimate could be obtained. The number of cases with 10 test and 10 control samples was based on the specifications of the ISO10993‐6 (International Organization for Standardization, 2016). This study was therefore carried out as an exploratory study.

### 2.7 Randomization

A randomization list was created for the overall study that included 60 patients (Institute of Epidemiology and Medical Biometry, University of Ulm, Germany). Assignment to the various groups was made in six layers. The data were stratified as follows:

- By sex (two groups: male or female)
- By region of the test tooth (three groups: anterior, premolar, and molar)

The study director or a person authorized by him instructed the treatment center by fax as to the type of treatment to be performed according to the randomization list.

### 2.8 Blinding

The laboratory received the samples in an anonymous form. The results were recorded on dedicated forms. The blinding was maintained until the samples had been completely prepared, analyzed, documented, and taken to a different place with a different operator than the researcher responsible for the histomorphological evaluation.

### 2.9 Statistical analysis

For the metric target variables, the minimum, median, and maximum were reported. Nominal and ordinal features were described with their absolute and relative frequencies.

Differences between the test and control groups were tested using the Wilcoxon rank‐sum test. Given the exploratory nature of this study, all statistical results are hypothetical in nature and should not be interpreted as confirmatory. All statistical tests were carried out at level *α* = .05 (two sided). No adjustment was made for multiple testing. The statistical analysis was performed with SAS® Version 9.4 and IBM SPSS Statistics 21.

Estimated sample size, randomization, blinding, and statistical analysis followed the same procedure as described in the previously published article of Schnutenhaus, Götz, et al. (Schnutenhaus, Götz, et al., 2018).

## 3 RESULTS

All patients were treated according to the clinical protocol. There were no postoperative complications. All included patients completed the study. The examinations took place in the period between June 4 and December 3, 2013. The study included 10 female patients and 10 male patients. The mean age of the patients was 46.6 (21.9–71.4) years. In the test group, the mean age was 44.3 (21.9–71.4) years; in the control group, it was 48.8 (33.1–58.3) years. The randomized distribution of the teeth is shown in Table 2. Results of the study population have been previously published by the first author (Schnutenhaus, Götz, et al., 2018).

**Table 2:** Distribution of teeth by region

Region	Test group	Control group	Total
Anteriors	5	7	12
Premolars	4	2	6
Molars	1	1	2

### 3.1 Semiquantitative histological, histochemical, and immunohistochemical analysis

Besides the histomorphometric analysis, the 20 histological samples were histochemically and immunohistochemically evaluated for the identification of bone metabolism and vascularization factors.

The results are presented in Table 3. The Wilcoxon rank‐sum test showed no significant difference between test and control groups for any parameter. According to the descriptive data, the use of a combination material seems to result in slightly higher values of Runx2 and VWF.

**Table 3:** Semiquantitative evaluation by minimum, median, and maximum, as well as by the statistical test (Wilcoxon rank‐sum test)

Parameters	Procedure	Valid records	Minimum	Median	Maximum	Hypothesis test
Infiltrate	ARP	10	0	1	2	0.61
	Control	10	0	1	3	
Tartrate‐resistant acid phosphatase	ARP	10	0	1	3	0.40
	Control	10	0	1	2	
ED1	ARP	10	0	1	3	0.93
	Control	9	0	1	2	
Alkaline phosphatase	ARP	10	0	1	3	0.78
	Control	9	0	1	3	
Runt‐related transcription factor 2 (Runx2)	ARP	10	0	2	3	0.29
	Control	10	0	0.5	2	
von Willebrand factor	ARP	10	2	2	3	0.37
	Control	10	1	2	2	
Collagen Type I	ARP	10	1	2	3	0.94
	Control	10	0	2	3	
Osteocalcin	ARP	10	0	2	3	0.53
	Control	10	0	1.5	3	
Osteopontin	ARP	10	0	2	3	0.78
	Control	10	0	1	3	

*Note*. The following values: 0 = *none*; 1 = *low*; 2 = *pronounced*; 3 and 4 = *very pronounced* have been assigned to the different levels.

Abbreviation: ARP, alveolar ridge preservation.

## 4 DISCUSSION

The histological, histochemical, and immunohistochemical examination of 20 biopsies revealed no significantly different results between the test (ARP) group and the control group. Descriptive data showed trends that could be of clinical relevance. The sample collection violates the manufacturer's drill protocol; thus, the surgical concept of this kind of study is problematic. Additionally, histological examinations are related to a high effort.

The histological examinations performed at different times make it more difficult to compare various studies that include observation periods between 3 (Heberer et al., 2011; Ruga, Gallesio, Chiusa, & Boffano, 2011; Serino, Rao, Iezzi, & Piattelli, 2008) and 9 months (Brkovic et al., 2012). Barone et al. (Barone et al., 2008), for example, showed significant differences of the mineralization process after 7 months between ARP with xenografts and extraction sockets with no intervention. In a review of De Risi et al. (De Risi et al., 2015), no significant effects of timing, surgical procedure, or material used on histological parameters of ARP measures could be found. The histological effects of ARP measures were mostly examined 3 months after tooth extraction (MacBeth et al., 2017). This is in line with the protocol of delayed immediate implant placement, as prescribed for the present study. After 3 months, significant differences of the mineralization process are not to be expected due to the chronological sequence of the regenerating mechanisms (Trombelli et al., 2008).

Furthermore, a direct comparison with different studies is hardly possible because of high variation in treatment protocols and materials used as well as in histological analyzing methods applied for socket healing evaluation (MacBeth et al., 2017). It must be stated that many of the studies including histological examinations contain an insufficient number of patients and therefore should rather be considered case reports (Araujo & Lindhe, 2005; Engler‐Hamm, Cheung, Yen, Stark, & Griffin, 2011; Kesmas et al., 2010). Another limitation is that the results of ARP measures are often not compared to unassisted socket healing (Checchi, Savarino, Montevecchi, Felice, & Checchi, 2011; Hoang & Mealey, 2012; Mardas, Chadha, & Donos, 2010; Margonar et al., 2012; Scheyer et al., 2016; Wood & Mealey, 2012). An evaluation of ARP at clinical and histomorphometric levels can take place if a control group with untreated extraction sockets is available along with the test group for comparative evaluation.

Despite the differences in the biomaterials and treatment methods, various systematic reviews show positive clinical results after ARP (Majzoub, Ravida, Starch‐Jensen, Tattan, & Suárez‐López del Amo, 2019; Stumbras, Kuliesius, Januzis, & Juodzbalys, 2019). At the histomorphometric level, the results regarding newly formed bone are inconsistent and depend on the biomaterial (Barallat et al., 2014; Canellas et al., 2019). It can be observed that materials that have an osteoinductive effect are superior to the placeholders with bone substitute materials. However, this has to be confirmed with further studies (Canellas et al., 2019; Pranskunas, Galindo‐Moreno, & Padial‐Molina, 2019).

Further on, the histologic results are influenced by patient selection. In patients who have a periodontal disease, new bone formation takes more time and is less predictable than in patients without a preexisting periodontitis (Ahn & Shin, 2008). The age of the patient is another influence on the healing process after tooth extraction, as angiogenesis and osteogenesis are delayed in aging patients (Nahles et al., 2013). In the present study, the patients of the control group were 4.5 years older than those of the test group. An influence of the age difference on histological findings cannot be excluded. Due to the study protocol and the number of patients that is too small for further differentiation, no assessment can be made in the present evaluation of the influence of general health factors. For this reason, no influences of the reason for the extraction, for example, trauma or periodontitis, are described.

It is not possible to differentiate the effects of socket grafting from the effects of socket sealing with a membrane. The use of membranes as a barrier preventing epithelial cells to migrate into the extraction socket is discussed controversially (Horowitz et al., 2012). Different investigations have shown that the use of membranes resulted in higher bone regeneration (Perelman‐Karmon, Kozlovsky, Liloy, & Artzi, 2012; Troiano et al., 2018) or a reduced resorption of the coronal bone (Caneva et al., 2010), whereas a recently published study showed no significant influence of membranes on the healing process (Mandarino, Luz, Moraschini, Rodrigues, & Barboza, 2018). Faria‐Almeida et al. (Faria‐Almeida, Astramskaite‐Januseviciene, Puisys, & Correia, 2019) come to the conclusion in their review that the use of membranes reveals to archives to better results.

It was expected that different factors of new bone formation would be found with the semiquantitative histological characterization of samples introduced in the present study. The determination of the proportional bone distribution, soft tissue, or residual graft material, which was not expected due to the material used, is possibly no evidence of the clinical relevance of characteristic values.

The fact that a comparison with other studies is hardly possible because of abovementioned reasons is even intensified when comparing histochemical and immunohistochemical results only.

In a study of Brkovic et al. (Brkovic et al., 2012), immunohistochemistry was performed on five bone samples. Osteonectin, a phosphorylated, noncollagenous glycoprotein which is thought to be one regulator of bone metabolism, was detected in osteoblasts and osteoblast‐like cells in two test groups, which included samples having been extracted at healed extraction sites 9 months after tooth removal. The authors assumed that remodeling of bone at the grafted sites was still ongoing (Brkovic et al., 2012). It has to be said that there was no control group for comparison without intervention after tooth extraction. The present study also showed the expression of proteins (Runx2, collagen, OC, and OP) in both groups. Immunoreactivity of OC, OP, and Runx2 was expressed slightly more pronounced in the test group, but no significant differences were found. Similar results were found in another study by Milani et al. (Milani et al., 2016), where higher expression of anabolic and catabolic bone markers was found in extraction sockets that were grafted with deproteinized bovine bone compared to sockets with no intervention.

According to the descriptive data, the use of a collagen combination material seems to result in slightly higher values of VWF. Biological processes during wound/socket healing could be positively influenced by the trend towards more vascularization with the use of such a collagen material. The collagen material used in this study had no negative impact on bone healing. There was no evidence of increased inflammation compared to the control group. The accumulation of an allogenic combination material consisting of calcium sulfate with a bovine bone substitute increased vital bone, probably because of a higher vascularization in the less dense material (Vance et al., 2004). The stimulation of angiogenesis by appropriate measures could support osteogenesis being dependent on sufficient vascularization. Older patients with reduced angiogenesis might profit from the positive effect of ARP measures (Nahles et al., 2013).

However, with the methods employed in the present study, it can be concluded that the combination material has no proven influence or clinical relevance on the formation of new bone, neither can a possible influence be disproved. However, the clinical evaluation according to ARP showed a significant volume preservation in the buccal aspect of the alveolus with the collagen material used (Schnutenhaus, Doering, Dreyhaupt, Rudolph, & Luthardt, 2018). A potential advantage of delayed implant placement after a healing period of more than 3 months when performing ARP needs to be assessed in further investigations.

For studies with immunohistochemical targets, sample‐size estimates might be of interest.

## AUTHOR CONTRIBUTIONS

S. I. S. made contributions to the acquisition of funding, conception, and design, as well as substantial contribution to the coordination of the study, clinical performance, acquisition of data, and drafting the manuscript. C. E. made contributions to the conception and design of the study and was involved in drafting.

J. D. made contributions to the conception and statistical design of the study and was involved in drafting. H. R. and R. L. made substantial contribution to the conception, design, and coordination of the study and were involved in drafting the manuscript. W. G. made contributions to conception and design, as well as substantial contribution to the coordination to the coordination of the study, clinical performance, acquisition of data, histological examination, and drafting the manuscript. All authors read and approved the final manuscript.
